# Enhanced Efficacy of Ciprofloxacin and Tobramycin against *Staphylococcus aureus* When Combined with Corydalis Tuber and Berberine through Efflux Pump Inhibition

**DOI:** 10.3390/antibiotics13050469

**Published:** 2024-05-20

**Authors:** Yena Seo, Minjun Kim, Tae-Jong Kim

**Affiliations:** 1 Department of Forest Products and Biotechnology, Kookmin University, Seoul 02707, Republic of Korea; yna127@kookmin.ac.kr (Y.S.); ykmj111@kookmin.ac.kr (M.K.); 2 Forest Carbon Graduate School, Kookmin University, Seoul 02707, Republic of Korea

**Keywords:** berberine, Corydalis Tuber extract, efflux pump inhibitors, *Staphylococcus aureus*

## Abstract

One way that bacteria develop antibiotic resistance is by reducing intracellular antibiotic concentrations through efflux pumps. Therefore, enhancing the efficacy of antibiotics using efflux pump inhibitors provides a way to overcome this type of resistance. Notably, an increasing number of pathogenic *Staphylococcus aureus* strains have efflux pump genes. In this study, the extract from *Corydalis ternata* Nakai tuber (Corydalis Tuber) at 512 mg/L was demonstrated to have an antibiotic synergistic effect with ciprofloxacin at 2 mg/L and tobramycin at 1024 mg/L against methicillin-resistant *S. aureus* (MRSA). Berberine, an isoquinoline alkaloid identified in Corydalis Tuber, was identified as contributing to this effect. Ethidium bromide efflux pump activity assays showed that Corydalis Tuber extract and berberine inhibited efflux, suggesting that they are efflux pump inhibitors. Molecular docking simulations suggested that berberine binds to *S. aureus* efflux pump proteins MepA, NorA, NorB, and SdrM. Additionally, berberine and Corydalis Tuber extract inhibit biofilm formation, which can confer antibiotic resistance. This study’s findings suggest that Corydalis Tuber, a traditional herbal medicine, and berberine, a medicinal supplement, act as *S. aureus* efflux pump inhibitors, synergistically increasing the efficacy of ciprofloxacin and tobramycin and showing promise as a treatment for antibiotic-resistant *S. aureus* infections, including MRSA.

## 1. Introduction

Antibiotics are used to ameliorate bacterial infections. However, the continuous use of antibiotics selects for resistant strains, leading to the emergence of antimicrobial resistance [1]. Bacteria have evolved various ways to survive antibiotic treatments [2]. These include modifying the antibiotic’s target proteins, deactivating the antibiotics, and lowering the intracellular antibiotic levels through drug efflux pumps [3,4]. By maintaining antibiotic levels below the minimum inhibitory concentration inside bacteria cells, for example, through drug efflux pumps, is one way bacterial strains can achieve drug resistance [5,6]. Therefore, inhibiting these pumps has been proposed as a strategy to combat infections caused by multidrug-resistant bacteria [6,7].

In a comprehensive analysis of blood infections spanning 45 countries and 20 years (1997 to 2016) and involving 264,901 pathogenic strains, *Staphylococcus aureus* strains emerged as the predominant pathogens, constituting 20.7% of cases [8]. Moreover, a study conducted in 2009 examined 563 *S. aureus* strains from patients with blood infections and revealed that 39.8% harbored at least one drug efflux pump gene [7]. These findings underscore the prevalence of drug efflux pumps in infectious *S. aureus* strains and highlight their significance as therapeutic targets. Efflux pumps of *S. aureus* typically belong to the major facilitator superfamily and the multidrug and toxic compound extrusion [6,9,10]. Notably, previous research has demonstrated that the alkaloid reserpine binds to *S. aureus*’ MepA efflux pump, inducing structural changes and impairing its ability to efflux antibiotics [11]. Therefore, the identification of chemical compounds capable of binding to these efflux pumps and thereby inhibiting their drug-exporting function will be a promising avenue for combating antibiotic-resistant bacterial infections.

Several previous studies have shown the synergistic activities of antibiotics and active chemical compounds in natural products [12,13,14,15]. For example, Ricini Semen extract and its active compound ricinoleic acid have been demonstrated to inhibit the expression of *mecA*, the gene encoding a penicillin-binding protein, showing synergistic effects with oxacillin [12]. Epigallocatechin gallate has been shown to disrupt the integrity of the cell wall of *S. aureus* and demonstrated synergistic activities with β-lactam antibiotics [16]. Moreover, several plant extracts and essential oils have been reported to act as inhibitors of drug efflux pumps [17]. For example, capsaicin, a major component of chili peppers, was found to act as an inhibitor of the NorA drug efflux pump in *S. aureus* [18]. Similarly, a molecular docking simulation study suggested that carvacrol and thymol bind to the NorA drug efflux pump [19].

Corydalis Tuber, the tuber of *Corydalis ternata* Nakai, is known to exhibit antibacterial, anti-inflammatory, and anticancer activities [20,21]. The active compounds of Corydalis Tuber are mainly alkaloids, including berberine [22], corydaline [23], and tetrahydropalmatine [22,24]. Berberine has shown antibacterial activity against the Gram-negative bacterium *Escherichia coli* and the Gram-positive bacteria *Bacillus subtilis* and *S. aureus* [25,26]. Its main antimicrobial mechanism is suggested to be the inhibition of the activity of the cell division protein FtsZ [27]. In addition, berberine has low toxicity to humans when used at conventional doses [28].

The aim of this study was to identify natural compounds from edible plants that can counteract antibiotic resistance in methicillin-resistant *S. aureus* (MRSA) by inhibiting antibiotic efflux pumps. To achieve this, a library of 392 plant methanolic extracts was searched to find those compounds to increase the susceptibility of two MRSA strains isolated from patients, MRSA CCARM 3820 and MRSA CCARM 3879, to antibiotics. Corydalis Tuber extract was identified and subsequently evaluated in combination with ciprofloxacin and tobramycin for its synergistic bactericidal activity against MRSA ATCC 33593, whose full genomic DNA sequence has been reported.

## 2. Results and Discussion

### 2.1. Corydalis Tuber Extract and Berberine Synergistically Enhanced the Bactericidal Activity of Ciprofloxacin and Tobramycin

A library of 392 plant methanol extracts using edible plants, including herbal medicines, in our laboratory was searched for those that enhanced the susceptibility of MRSA strains CCARM 3820 and CCARM 3879 to oxacillin (Supplementary Materials, Figure S1). Among them, 35 extracts inhibited MRSA’s cell growth by more than 80% with 4 mg/L of oxacillin, which was 12.8% of the minimum inhibitory concentration (Supplementary Materials, Table S1). Of these, we found no previous study on the bactericidal activity of five extracts against MRSA, including Alpiniae Oxyphyllae Fructus, Corydalis Tuber, Lepidii seu Descurainiae Semen, Linderae Radix, and Thujae Semen (Supplementary Materials, Table S1). These five ethanolic extracts were evaluated for their ability to enhance the susceptibility of two MRSA strains to oxacillin (Supplementary Materials, Table S2). Two extracts, Corydalis Tuber and Linderae Radix, produced more than 90% growth inhibition against both MRSA strains with 4 mg/L of oxacillin (Supplementary Materials, Table S2). Therefore, Corydalis Tuber extract was selected for further evaluation of its ability to synergize with three antibiotics, ciprofloxacin, oxacillin, and tobramycin (Table 1), each of which has a different mechanism of action. Ciprofloxacin inhibits cell division by interfering with DNA gyrase and topoisomerase IV, oxacillin inhibits bacterial cell wall synthesis, and tobramycin inhibits protein synthesis by binding to bacterial ribosomes [29]. For these experiments, *S. aureus* ATCC 33593, an MRSA strain, was used as its complete genome sequence is available (https://genomes.atcc.org/genomes/5940add780854658?tab=annotations-tab accessed on 1 March 2024).

Corydalis Tuber extract showed synergistic antibacterial activity with ciprofloxacin and tobramycin. This synergism was shown even when the concentrations of ciprofloxacin and tobramycin were reduced 8-fold (Table 1). However, in the case of oxacillin, which showed synergism with Corydalis Tuber extract activity during the screening process, only partial synergistic antibacterial activity was observed. Thus, while Corydalis Tuber extract greatly enhanced the activity of ciprofloxacin and tobramycin, its mechanism of action with oxacillin may differ, explaining the reduced synergistic effect.

In previous studies, alkaloid-based compounds have shown synergistic effects with antibiotics against *E. coli* [30] and *S. aureus* [31]. The chemical components of Corydalis Tuber extract are mainly alkaloids, such as berberine, corydaline, and tetrahydropalmatine [22,32,33], which were evaluated for their synergistic effects against *S. aureus* ATCC 33593 using ciprofloxacin, oxacillin, and tobramycin. Berberine showed synergistic activity with ciprofloxacin and tobramycin (Table 1). The antibiotic-enhancing activity of berberine was similar to that of Corydalis Tuber extract. Therefore, it is proposed that berberine contributed to Corydalis Tuber extract’s synergistic antibiotic effects. The HPLC analysis showed that the Corydalis Tuber extract contained 0.41% berberine (Supplementary Materials, Figure S2). This result is similar to those reported in previous studies, where the berberine content of Corydalis Tuber ethanol extract was 0.75% [34,35].

### 2.2. Time–Kill Curve Analyses Demonstrate Strong Synergistic Antibacterial Activity

**Figure 1:**
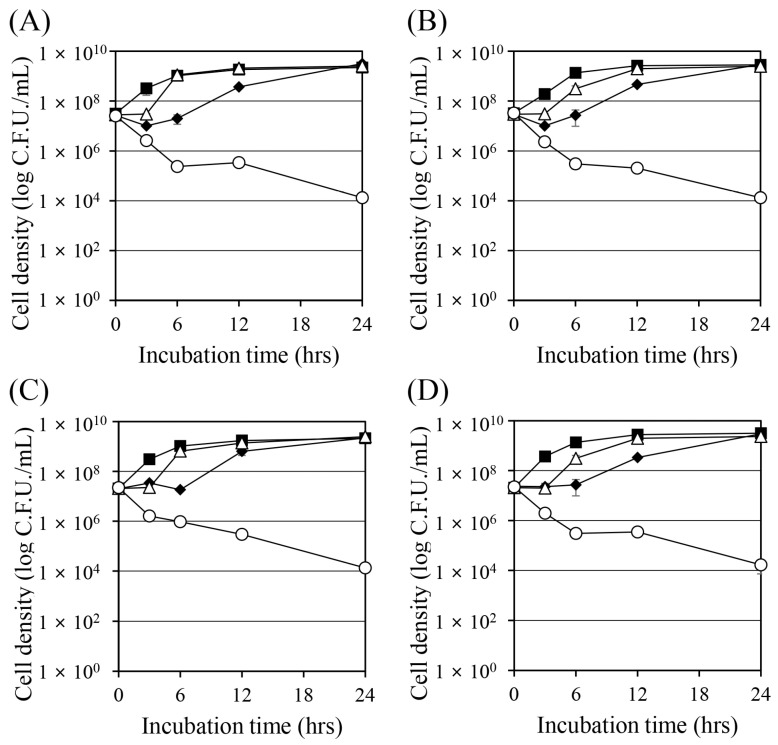
Time–kill curves of *Staphylococcus aureus* ATCC 33593 when challenged with ciprofloxacin, tobramycin, Corydalis Tuber extract, and berberine. Cell density was measured by colony-forming units. (**A**) Ciprofloxacin (0.25 mg/L) and Corydalis Tuber extract (64 mg/L) were added to the culture alone or in combination: control (no chemicals added; ■), ciprofloxacin (◆), Corydalis Tuber extract (△), and ciprofloxacin and Corydalis Tuber extract (○). (**B**) Ciprofloxacin (0.25 mg/L) and berberine (64 mg/L) were added to the culture alone or in combination: control (■), ciprofloxacin (◆), berberine (△), and ciprofloxacin and berberine (○) (**C**) Tobramycin (128 mg/L) and Corydalis Tuber extract (128 mg/L) were added to the culture alone or in combination: control (■), tobramycin (◆), Corydalis Tuber extract (△), and tobramycin and Corydalis Tuber extract (○). (**D**) tobramycin (256 mg/L) and berberine (64 mg/L) were added to the culture alone or in combination: control (■), tobramycin (◆), berberine (△), and tobramycin and berberine (○). The experiments were performed in triplicate and expressed as the mean and standard deviation.

Time–kill curve experiments were performed to observe the changes in cell density when Corydalis Tuber extract or berberine was used together with ciprofloxacin and tobramycin (Figure 1). All substances were treated alone below their MIC, which resulted in cell growth over time. However, the combinations of ciprofloxacin and Corydalis Tuber extract (open circle in Figure 1A) and ciprofloxacin and berberine (open circle in Figure 1B) showed greater than 99.9% bactericidal activity after 24 h. Similarly, combinations of tobramycin and Corydalis Tuber extract (open circle in Figure 1C) and tobramycin and berberine (open circle in Figure 1D) showed strong bactericidal effects. These results indicate that the synergistic concentrations of the combined substances have bactericidal and not bacteriostatic activity.

### 2.3. Corydalis Tuber Extract and Berberine Are Efflux Pump Inhibitors

**Figure 2:**
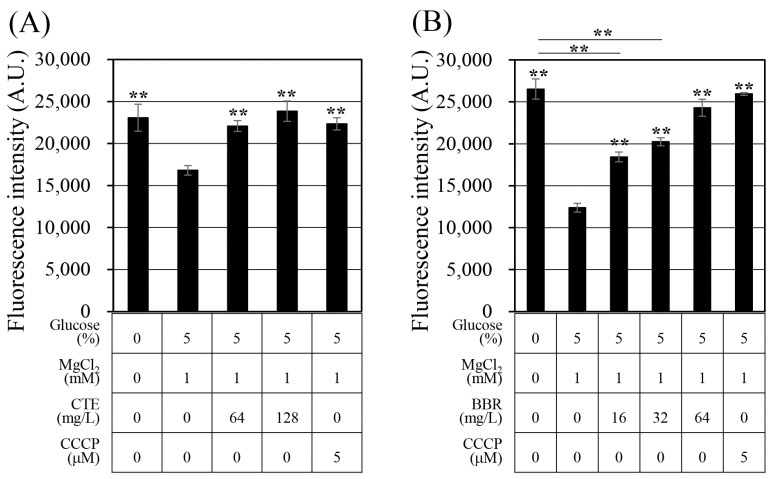
Evaluation of efflux function in *Staphylococcus aureus* ATCC 33593 with (**A**) Corydalis Tuber extract or (**B**) berberine. Fluorescence of ethidium bromide, in arbitrary units (A.U.), was measured after 10 min of treatment with each substance, Corydalis Tuber extract (CTE), carbonyl cyanide 3-chlorophenylhydrazone (CCCP), and berberine (BBR), the concentrations of which are shown below the graph. The experiments were performed in triplicate and expressed as the mean and standard deviation. Statistically significant differences among the means were identified using Student’s *t*-test in Microsoft Excel: two asterisks indicate *p* < 0.01.

Previous studies have suggested that aminoglycoside and fluoroquinolone antibiotics can be removed from bacterial cells via efflux pumps [36,37]. The antibiotics that showed synergistic effects with Corydalis Tuber extract and berberine in this study were tobramycin, an aminoglycoside antibiotic, and ciprofloxacin, a fluoroquinolone antibiotic. Ethidium bromide (EtBr) is a substrate of various efflux pumps and is frequently used to evaluate their activity [38,39]. Therefore, it was used to measure the change in the efflux pump activity in this study (Figure 2). Treatment with Corydalis Tuber extract prevented the efflux of EtBr from the cell, and berberine had a similar effect. The efflux pump inhibition activity of Corydalis Tuber extract and berberine was concentration-dependent. The inhibitor carbonyl cyanide 3-chlorophenylhydrazone—which inhibits efflux pump activity by removing the proton motive force [40], thereby inhibiting EtBr efflux from the cells—was also tested as a positive control.

Berberine has previously been reported to inhibit the efflux pumps of *E*. *coli* [41] and *Pseudomonas aeruginosa* [42]. This study’s results confirm a similar synergistic effect in the Gram-positive bacterium *S. aureus*. The *S. aureus* ATCC 33593 contains four genes (*mepA*, *norA*, *norB*, and *sdrM*) encoding antibiotic efflux pumps; MepA is known to efflux aminoglycosides and fluoroquinolone antibiotics [3,43]. Tobramycin is an aminoglycoside, and it has been suggested that tobramycin is effluxed by MepA. In contrast, NorA, NorB, and SdrM are reported to efflux fluoroquinolone antibiotics [3]. Ciprofloxacin is a fluoroquinolone antibiotic, and it has been suggested that MepA, NorA, NorB, and SdrM efflux ciprofloxacin. This study’s results demonstrate that Corydalis Tuber extract and berberine inhibited the efflux of EtBr, suggesting this as a mechanism for their synergistic effect with ciprofloxacin and tobramycin.

### 2.4. Berberine Interacts with Efflux Pump Proteins and the Protein PBP2a

**Figure 3:**
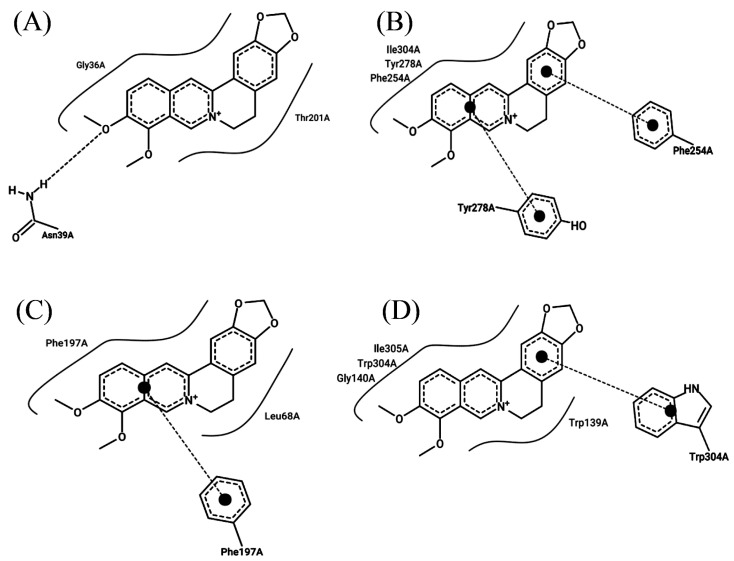
Molecular docking analysis of berberine with the efflux pump proteins of *Staphylococcus aureus* ATCC 33593: (**A**) MepA, (**B**) NorA, (**C**) NorB, and (**D**) SdrM. The curved line around berberine indicates hydrophobic interactions, the dotted line between oxygen atom and hydrogen atom in (**A**) shows a hydrogen bond, and the dotted lines with a large dot at the end in (**B**–**D**) denote stacking interactions.

To demonstrate the mechanism of berberine’s efflux pump inhibition, a molecular docking simulation analysis of efflux pump proteins was performed (Supplementary Materials, Table S3). The binding assay between the alkaloid constituents of the Corydalis Tuber extract and the four efflux pump proteins of *S. aureus* showed that berberine had strong binding affinities with the proteins, with −7.9, −7.6, −7.8, and −9.4 kcal/mol for MepA, NorA, NorB, and SdrM, respectively. A previous study reported that berberine interacted with AdeB, an *Acinetobacter bauamii* efflux pump protein, with a binding affinity of −7.42 kcal/mol [44]. In addition, this study’s protein–ligand interaction analysis showed that hydrophobic and stacking interactions played a primary role in the stability of the binding (Figure 3), which is consistent with the results reported by previous studies [41,45].

Furthermore, it has been previously reported that berberine synergistically enhances the bactericidal activity of oxacillin [46]. In contrast, berberine and oxacillin exhibited only partial synergism in this study (Table 1). In addition, it has been suggested that berberine binds to PBP2a, a protein that confers resistance to oxacillin [47]. Therefore, molecular docking simulations of berberine were performed using the PBP2a protein (PDB code: 4CJN) in a previous study [48] and the PBP2a protein from *S. aureus* ATCC 33593 (Supplementary Materials, Figure S3). The binding affinity was −7.9 kcal/mol for PBP2a 4CJN and −7.0 kcal/mol for the PBP2a protein of *S. aureus* ATCC 33593. The decreased binding affinity for the PBP2a protein of strain ATCC 33593 may explain why berberine showed only partial synergy (Table 1). To determine the reason for this difference in binding affinities, the amino acid sequences of the two PBP2a proteins were compared (Supplementary Materials, Figure S4). In the PBP2a from *S. aureus* ATCC 33593, amino acid 204, asparagine in strain Mu50’s PBP2a 4CJN, was substituted with lysine, and amino acid 246, glycine in Mu50’s PBP2a 4CJN, was substituted with glutamic acid. These introductions of lysine and glutamic acid, strongly charged amino acids close to the hydrophobic pocket where berberine binds, likely induce modifications to the berberine binding pocket, resulting in a lower binding force (Supplementary Materials, Figure S3). Thus, this study suggests that the interfering effect of PBP2a on oxacillin may not have been sufficiently inhibited by berberine in *S. aureus* ATCC 33593.

### 2.5. Corydalis Tuber Extract and Berberine Reduce Biofilm Formation

**Figure 4:**
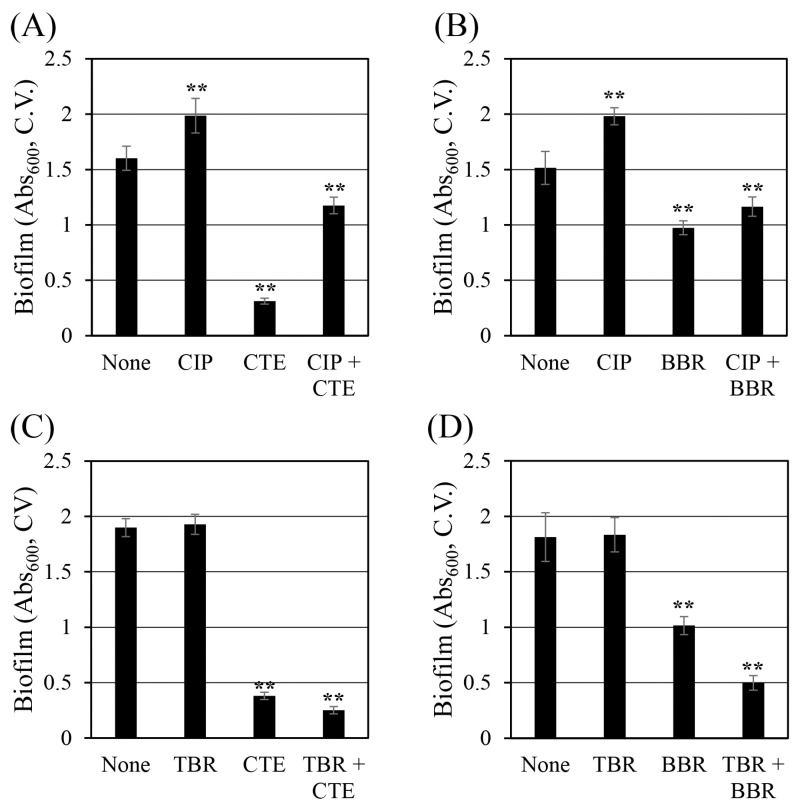
Changes in the biofilm formation of *Staphylococcus aureus* ATCC 33593 by (**A**) ciprofloxacin (CIP, 0.25 mg/L) and Corydalis Tuber extract (CTE, 64 mg/L), alone and in combination; (**B**) ciprofloxacin (0.25 mg/L) and berberine (BBR, 64 mg/L), alone and in combination; (**C**) tobramycin (TBR, 128 mg/L) and Corydalis Tuber extract (128 mg/L), alone and in combination; and (**D**) tobramycin (256 mg/L) and berberine (64 mg/L), alone and in combination. Six replicates of each experiment were performed, and the results are expressed as the means and standard deviations. Statistically significant differences among the means were identified using Student’s *t*-test in Microsoft Excel: two asterisks denote *p* < 0.01.

Biofilms are masses of organic matter composed of cells with extracellular, sticky polymers that interfere with the penetration of antibiotics, reducing their effectiveness in treating infectious diseases [49,50]. Therefore, inhibiting biofilm formation is a way to increase the bactericidal effect of antibiotics. In this study, the effects of Corydalis Tuber extract and berberine on the ability of *S. aureus* (Figure 4) to form biofilms were evaluated. Corydalis Tuber extract and berberine reduced biofilm formation by 81% (Figure 4A) and 36% (Figure 4B), respectively. When ciprofloxacin was combined with Corydalis Tuber extract and berberine, biofilm formation was reduced by 40% and 41%, respectively, compared to ciprofloxacin alone. Surprisingly, ciprofloxacin significantly increased biofilm formation. A recent study proposed that ciprofloxacin at sub-minimal inhibitory concentrations (MICs) increased the biofilm formation of *S. aureus* by increasing the synthesis of polysaccharide intercellular adhesion through an *agrC*-dependent mechanism [51]. Similarly, in this study, ciprofloxacin increased the biofilm formation of *S. aureus* by 24% and 31% compared to ethanol and dimethyl sulfoxide (DMSO) controls, respectively. Therefore, the presence of ciprofloxacin partially offset the biofilm inhibitory ability of Corydalis Tuber extract and berberine.

Tobramycin at 128 mg/L (1/8 MIC) did not change the biofilm formation of *S. aureus*. In contrast, Corydalis Tuber extract and berberine reduced biofilm formation by 80% (Figure 4C) and 44% (Figure 4D), respectively. When Corydalis Tuber extract or berberine was combined with tobramycin, biofilm formation was reduced by 87% and 73%, respectively, compared to the control. Unlike the results seen with ciprofloxacin, these reductions suggest the possibility of a synergistic inhibition of biofilm formation by *S. aureus* with tobramycin.

Furthermore, a previous study suggested that berberine’s inhibitory activity on the biofilm formation of *S. aureus* was achieved by inhibiting the formation of amyloid fibrils, which are composed of phenol-soluble modulins [52]. In addition, berberine is reported to inhibit the expressions of *cidA*, *icaA*, and *sarA*, which are genes related to biofilm formation [53]. The inhibitory effect of Corydalis Tuber extract on biofilm formation cannot be explained solely by the inhibition of biofilm formation by berberine. Rather, the findings suggest the presence of other active chemicals in the extract that inhibit the biofilm formation of *S. aureus*.

## 3. Materials and Methods

### 3.1. Staphylococcus aureus Strains and Culture Conditions

The *S. aureus* ATCC 33593 strain was purchased from the Korean Culture Center of Microorganisms (Seoul, Republic of Korea). The MRSA CCARM 3820 and CCARM 3879 strains were purchased from the Culture Collection of Antimicrobial Resistant Microbes (CCARM, Guri, Republic of Korea). All *S. aureus* strains were stored at −80 °C in 25% glycerol (catalog number: 4066–4400, Daejung Chemical & Metals Co., Ltd., Siheung, Republic of Korea). Cells were cultured using tryptic soy broth (TSB, catalog number: 211825, Becton Dickinson Korea Co., Ltd., Seoul, Republic of Korea) and tryptic soy agar (TSA) made using TSB with 1.5% agar (catalog number: 214010, Becton Dickinson Korea Co., Ltd.). Cultures were incubated at 37 °C with a shaking speed of 250 rpm. A saline solution was prepared by dissolving sodium chloride (catalog number: S0476, Samchun Chemicals Co., Ltd., Seoul, Republic of Korea) at 0.85% (*w*/*v*). Phosphate-buffered saline (PBS) was made with 8 g/L of sodium chloride, 0.2 g/L of potassium chloride (catalog number: PR-1938, Tedia Company Inc., Fairfield, OH, USA), 1.44 g/L of sodium phosphate dibasic (catalog number: 7613–4405, Daejung Chemical & Metals Co., Ltd.), and 0.245 g/L of potassium phosphate monobasic (catalog number: P1122, Samchun Chemicals Co., Ltd.). All solutions were autoclaved at 121 °C for 20 min.

### 3.2. Chemicals

The berberine chloride hydrate (catalog number: B0450), ciprofloxacin hydrochloride monohydrate (catalog number: C2227), and tobramycin (catalog number: T2503) were purchased from the Tokyo Chemical Industry Co., Ltd. (Tokyo, Japan). Oxacillin sodium salt (catalog number: sc-224180B) was purchased from Santa Cruz Biotechnology Inc. (Dallas, TX, USA). Carbonyl cyanide 3-chlorophenylhydrazone (CCCP, catalog number: B34950) was purchased from Invitrogen (ThermoFisher Scientific Korea Ltd., Seoul, Republic of Korea). Ethidium bromide (catalog number: L07482) was purchased from Alfa Aesar (ThermoFisher Scientific Korea Ltd.). The berberine chloride hydrate was dissolved in DMSO (catalog number: 000D0458, Samchun Chemicals Co., Ltd.), and the ciprofloxacin hydrochloride monohydrate, EtBr, oxacillin sodium salt, and tobramycin were dissolved in distilled water.

### 3.3. Preparing the Corydalis Tuber Extract

The Corydalis Tuber extract was prepared according to the method of a previous study [12]. Briefly, 15 g of dehydrated Corydalis Tuber, the tuber of *Corydalis ternata* Nakai, was crushed into 1 × 1 cm pieces, soaked in 150 mL of 95% ethyl alcohol (catalog number: 000E0219, Samchun Chemicals Co., Ltd.) and then extracted at 50 °C for 3 h, shaking every 30 min. The extract was filtered using Whatman™ qualitative filter paper Grade 1 (catalog number: 1002-110, Cytiva™, Sigma-Aldrich, Merck KGaA, Darmstadt, Germany), and the filtrate was concentrated using a rotary evaporator (RV-10, IKA Korea Ltd., Seoul, Republic of Korea). The concentrated extract was freeze-dried (FDU-2110, EYELA, SunilEyela Co., Ltd., Seongnam, Republic of Korea) for 3 days and dissolved in ethyl alcohol anhydrous (catalog number: 000E0243, Samchun Chemicals Co., Ltd.) before experiments. The final yield of Corydalis Tuber ethanol extract was 1.16%.

### 3.4. Analyzing the Berberine Concentration of the Corydalis Tuber Extract Using High-Performance Liquid Chromatography

The Corydalis Tuber extract was analyzed for its berberine content using high-performance liquid chromatography (HPLC) according to a previous study’s method [54]. The analysis was performed on an Arc HPLC (Waters Corporation, Milford, MA, USA.) coupled with a 2998 photodiode array detector using a YMC-Triart C18 column (catalog number: TA12S05-2546WT; YMC Korea, Seongnam, Republic of Korea). The mobile phase was water–acetonitrile at 1:1 (*v*/*v*), with 3.4 g/L potassium dihydrogen phosphate (catalog number: 169-04245) and 1.7 g/L of sodium dihydrogen phosphate (catalog number: 169-04245), both purchased from Fujifilm Wako Pure Chemical Corporation, Osaka, Japan, and 1.7 g/L of sodium lauryl sulfate (catalog number: 1436, Duksan Pure Chemicals Co., Ltd., Ansan, Republic of Korea). The HPLC analysis was carried out under isocratic conditions with a flow rate of 1 mL/min and a column temperature of 40 °C. Berberine was identified at a wavelength of 345 nm; the total analysis time was 60 min.

### 3.5. Evaluating the Synergistic Antibacterial Activity of Antibiotics and Corydalis Tuber Extract or Berberine

The evaluation of the MICs of the antibiotics ciprofloxacin and tobramycin, Corydalis Tuber extract, and berberine against the selected *S. aureus* strains was performed according to the method of a previous study [12]. Briefly, single colonies of the *S. aureus* strains streaked on TSA were selected and pre-cultured in 5 mL of TSB at 37 °C for 24 h. The pre-cultured cells were then inoculated into 5 mL of TSB to an optical density (OD) of 0.05 at a wavelength of 600 nm (OD_600_ = 0.05). The cells were incubated at 37 °C for 24 h. The minimum concentration of the substance at which *S. aureus* cells did not grow after 24 h was determined as the MIC. The MIC experiments were performed in triplicate.

The synergistic antibacterial activity of Corydalis Tuber extract and berberine with each antibiotic was evaluated for *S. aureus* ATCC 33593 by modifying a previous study’s method [55]. Pre-cultures were prepared and incubated under the culture condition previously described for MIC determination. Subsequently, the cultures were incubated with each antibiotic in combination with Corydalis Tuber extract or berberine at 37 °C for 24 h. The concentration of each compound in a combination at which inhibition of growth was observed was determined using sequential 2-fold dilutions from the MIC concentration of each substance. The combination with the lowest concentration of antibiotic was selected for evaluation. Using the selected combination, the synergistic antibacterial activity was evaluated using the fractional inhibitory concentration (FIC) index method [56,57,58]. The FIC_A_ is the MIC of compound A in the combination/MIC of compound A alone; likewise, the FIC_B_ is the MIC of compound B in the combination/MIC of compound B alone. The FIC index (FICI) is FIC_A_ + FIC_B_. An FICI ≤ 0.5 indicates synergistic antibacterial activity, 0.5 < FICI ≤ 1 indicates partial synergistic antibacterial activity, 1 < FICI ≤ 4 indicates indifference, and an FICI > 4 indicates antagonistic antibacterial activity. Each synergistic antibacterial activity experiment was performed in triplicate.

### 3.6. Measuring the Time–Kill Curves for S. aureus ATCC 33593 Treated with Antibiotics and Corydalis Tuber Extract or Berberine

To create the time–kill curves, the *S. aureus* strains were pre-cultured as previously described for MIC determination. Each antibiotic, with either Corydalis Tuber extract or berberine, was added to a main culture inoculated to an OD_600_ = 0.05. After incubation at 37 °C and 250 rpm for specified times (0, 3, 6, 12, and 24 h), the cells were diluted in saline, and the cell density was determined as colony-forming units on TSA. The experiments were performed in triplicate.

### 3.7. Evaluating the Efflux Pump Inhibition of Corydalis Tuber Extract and Berberine Using Ethidium Bromide

To assess whether Corydalis Tuber extract and berberine act as efflux pump inhibitors, the efflux activity was measured according to the study of Zmantar et al. [59], with a few modifications. Briefly, cells were pre-cultured as described for MIC determination. The *S. aureus* cells were inoculated to an OD_600_ = 0.05 and then incubated at 37 °C and 250 rpm for 3 h. Subsequently, the cells were harvested by centrifugation at 7820× *g* for 5 min and then resuspended in 5 mL of PBS. Then, 3 μg/mL of EtBr was added to the resuspended cells, followed by incubation at 21 °C and 250 rpm for 1 h to allow entry into the cells. After once again harvesting the cells by centrifugation at 7820× *g* for 5 min, they were resuspended in PBS with glucose at 5% and MgCl_2_ at 1 mM. Corydalis Tuber extract (64 mg/L or 128 mg/L), berberine (64 mg/L), and carbonyl cyanide 3-chlorophenylhydrazone (5 μM), used as a positive control, were added, and the cultures were incubated for 10 min at 21 °C. The fluorescence was measured at a wavelength of 587 nm by excitation at 550 nm with a QuantStudio™ 5 real-time polymerase chain reaction system (ThermoFisher Scientific Korea Ltd.) to evaluate the efflux of EtBr out of the cells.

### 3.8. Analyzing the Interaction of Efflux Pump Proteins with Berberine Using Molecular Dynamic Simulation

To evaluate whether berberine interacted with the efflux pump proteins of *S. aureus* ATCC 33593, molecular dynamics simulation analyses were performed. The efflux pump genes of *S. aureus* ATCC 33593 include *mepA*, *norA*, *norB*, and *sdrM*, and their structures have previously been predicted using AlphaFold 2 [60]. The chemical structure of berberine was downloaded from PubChem (https://pubchem.ncbi.nlm.nih.gov/ accessed on 1 March 2024), and the binding affinity was analyzed using AutoDock Vina 1.2.5 [61]. The grid dimensions were X = 60 Å, Y = 60 Å, and Z = 60 Å. The protein–ligand interaction was analyzed using ProteinPlus (https://proteins.plus/ accessed on 1 March 2024) [62].

### 3.9. Evaluating Biofilm Formation

For the quantitative analysis of biofilm formation, *S. aureus* cells were pre-cultured as for the MIC measurements. The main culture was prepared in TSB supplemented with glucose at 0.5%. Then, 100 μL of main cultures containing the various antibiotic, test substance, and combination treatments were dispensed into 96-well polystyrene microplates (catalog number: 34296, SPL Life Sciences Co., Ltd., Pocheon, Republic of Korea) and incubated at 37 °C for 24 h.

Biofilm formation was measured using the 1% crystal violet staining method [63,64]. The liquid culture, including planktonic cells, was removed and each well was rinsed three times using distilled water. Crystal violet (1%, 100 μL) was added to each well and incubated at room temperature for 15 min. The wells were again rinsed three times with distilled water. To release the stained crystal violet in biofilm, 100 μL of 95% ethanol was added to each well and incubated for 15 min at room temperature. The absorbance of each well was measured at 600 nm using a SynergyTM LX Multi-Mode Reader (BioTek, YBioTech Inc., Namyangju, Republic of Korea). The degree of inhibition of biofilm formation was calculated by comparing the amount of biofilm formed after treatment with each substance/combination to the amount of biofilm formed in the solvent control.

### 3.10. Statistical Analysis

Statistical analysis was performed using Student’s *t*-tests with the *t*-test function in Microsoft Excel 2019.

## 4. Conclusions

Corydalis Tuber extract and its active compound, berberine, synergistically enhanced the bactericidal activity of ciprofloxacin and tobramycin. Our study found that both Corydalis Tuber extract and berberine inhibited the efflux function of methicillin-resistant *Staphylococcus aureus* (MRSA), and molecular modeling indicated that berberine could bind to all four efflux pump proteins, MepA, NorA, NorB, and SdrM. This binding likely disrupts the efflux mechanism so that the antibiotic concentration in the cells is lower than the lethal level. This restores the bactericidal activity of antibiotics against MRSA. Furthermore, our study revealed that Corydalis Tuber extract and berberine exhibited inhibitory activities on *S. aureus* biofilm formation, which is crucial for combating persistent infections. These findings underscore the potential of Corydalis Tuber extract and berberine as adjunctive therapies with antibiotics for treating infectious diseases caused by antibiotic-resistant strains of *S. aureus*. Importantly, our research highlights the promise of natural compounds in enhancing the effectiveness of existing antibiotics against antibiotic-resistant bacterial infections, offering new strategies for combating the growing threat of antimicrobial resistance.

## 5. Patents

The results of this study have been used as a basis to apply for a Korean patent (application number: 10-2023-0093833; application date: 19 July 2023; patent title: Composition for enhancing antibiotic sensibility comprising Corydalis Tuber extract).

## Figures and Tables

**Table 1:** Bactericidal activity of three antibiotics and Corydalis Tuber extract and berberine against *Staphylococcus aureus* ATCC 33593 and evaluation of synergistic bactericidal activity by combinations of antibiotics and Corydalis Tuber extract or berberine.

Sample	MIC (mg/L)	Test Concentration for Synergy (mg/L)	FIC	FIC Index	Decision
Ciprofloxacin	2	0.25	0.125	0.25	Synergistic
Corydalis Tuber	512	64	0.125		
Ciprofloxacin	2	0.25	0.125	0.375	Synergistic
Berberine	256	64	0.25		
Oxacillin	128	8	0.0625	0.5625	Partial synergistic
Corydalis Tuber	512	256	0.5		
Oxacillin	128	8	0.0625	0.5625	Partial synergistic
Berberine	256	128	0.5		
Tobramycin	1024	128	0.125	0.375	Synergistic
Corydalis Tuber	512	128	0.25		
Tobramycin	1024	256	0.25	0.5	Synergistic
Berberine	256	64	0.25		

MIC: minimal inhibition concentration; FIC: fractional inhibitory concentration.

## Data Availability

The datasets used/analyzed during the current study are available from the corresponding author upon reasonable request.

## Supplementary Materials

The following supporting information can be downloaded at: https://www.mdpi.com/article/10.3390/antibiotics13050469/s1, Figure S1: Changes in the cell growth of two methicillin-resistant *Staphylococcus aureus* strains, CCARM 3820 (☐) and CCARM 3879 (■), after a 24 h treatment with 512 mg/L each of 392 methanolic plant extracts and 4 mg/L of oxacillin (1/8 MIC); Table S1: Methanolic extracts that inhibited the growth of the methicillin-resistant *Staphylococcus aureus* (MRSA) strains MRSA CCARM 3820 and MRSA CCARM 3879 by more than 80% when incubated at 512 mg/L with oxacillin (1/8 MIC, 4 mg/L) for 24 h; Table S2: Changes in the cell density of methicillin-resistant *Staphylococcus aureus* (MRSA) strains MRSA CCARM 3820 and MRSA CCARM 3879 after incubation with oxacillin (1/8 MIC, 4 mg/L) and five selected ethanolic extracts (512 mg/L) for 24 h; Figure S2: High-performance liquid chromatography chromatogram of the Corydalis Tuber ethanol extract. The arrow indicates the retention time of berberine; Table S3: Simulated molecular docking binding affinities of eight alkaloid compounds known to be in Corydalis Tuber extract to the four efflux pump proteins of *Staphylococcus aureus*; Figure S3: Molecular docking analysis with berberine and the PBP2A protein of *Staphylococcus aureus* ATCC 33593 (**A**) and *S. aureus* Mu50 (**B**; PDB code: 4CJN). The curved green lines indicate hydrophobic interactions and the black dotted line shows hydrogen bonds; Figure S4: The PBP2A protein amino acid sequences of *Staphylococcus aureus* ATCC 33593 and *S. aureus* Mu50. An asterisk indicates the presence of different amino acids at that position.
