# Supplementary material for: Enhanced Efficacy of Ciprofloxacin and Tobramycin against Staphylococcus aureus When Combined with Corydalis Tuber and Berberine through Efflux Pump Inhibition
Source: Antibiotics (Basel). 2024 May 20;13(5):469. doi: 10.3390/antibiotics13050469 (PMC11118900; doi:10.3390/antibiotics13050469)
Supplement: Supplementary file 1 [file antibiotics-13-00469-s001.zip › antibiotics-3000040-supplementary.pdf]

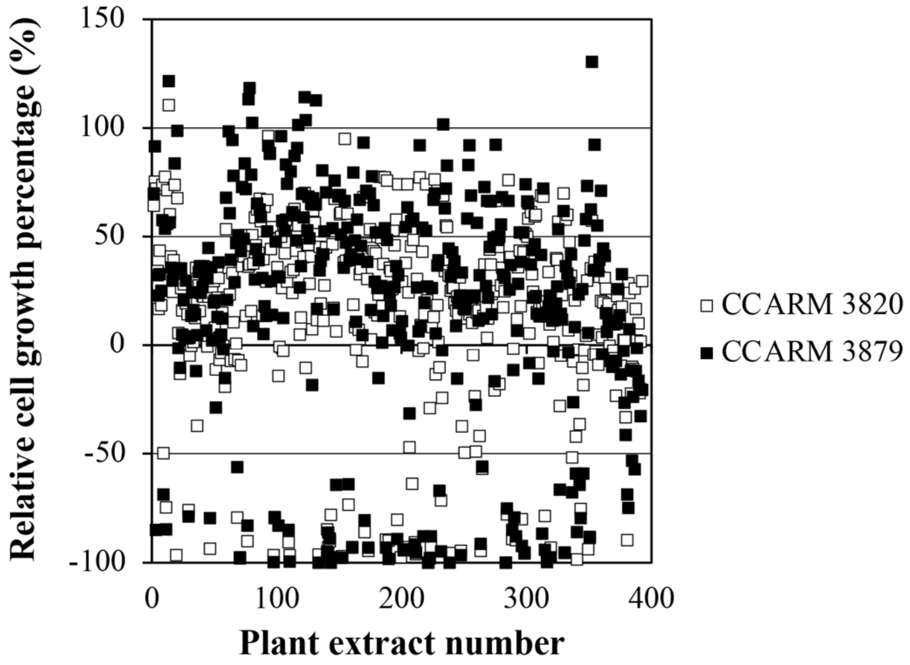

Supplementary Material Figure S1. Changes in the cell growth of two methicillin-resistant *Staphylococcus aureus* strains, CCARM 3820 (☐) and CCARM 3879 (■), after a 24 h treatment with 512 mg/L each of 392 methanolic plant extracts and 4 mg/L of oxacillin (1/8 MIC).

Supplementary Material Table S1. Methanolic extracts that inhibited the growth of the methicillin-resistant *Staphylococcus aureus* (MRSA) strains MRSA CCARM 3820 and MRSA CCARM 3879 by more than 80% when incubated at 512 mg/L with oxacillin (1/8 MIC, 4 mg/L) for 24 h.

Extract	MRSA CCARM 3820	MRSA CCARM 3879
	Relative cell growth (%)	Relative cell growth (%)
**Alpiniae Oxyphyllae Fructus**	**−94.59**	**−96.49**
Araliae Continentalis Radix	−85.90	−80.41
Cannabis Fructus	−90.52	−91.42
Cinnamomi Cortex	−96.34	−99.65
**Corydalis Tuber**	**−89.08**	**−85.00**
Crassirhizomae Rhizoma	−97.03	−97.77
Fruit of *Myrciaria dubia*	−93.05	−97.51
Impatientis Semen	−93.09	−92.66
Kaempferiae Rhizoma	−96.03	−95.34
Leaf of *Origanum vulgare*	−95.01	−99.77
Leaf of *Pinus densiflora*	−100	−100
Leaf of *Rosmarinus officinalis*.	−94.45	−92.99
Leaf of *Salvia officinalis*	−95.69	−95.12
Leaf of *Silybum marianum*	−88.49	−88.13
**Lepidii seu Descurainiae Semen**	**−90.55**	**−84.88**
**Linderae Radix**	**−95.50**	**−100**
Magnoliae Cortex	−89.51	−87.91
Mori Cortex Radicis	−97.88	−97.56
Ocimi Herba	−89.37	−92.95
Osterici Radix	−89.97	−88.87
Perillae Japonicae Semen	−95.38	−95.34
Perilliae Semen	−89.96	−82.91
Piperis Nigri Fructus	−96.15	−100
Psoraleae Semen	−92.81	−94.30
Pulvis Myristicae Seminis	−98.41	−85.87
Ricini Semen	−100	−100
Saposhnikoviae Radix	−89.85	−98.13
Sappan Lignum	−92.42	−87.78
Siegesbeckiae Herba	−84.60	−94.78
Sophorae Radix	−97.47	−97.80
Syzygii Flos	−96.57	−99.49
Terminaliae Fructus	−92.69	−86.12
**Thujae Semen**	**−80.15**	**−89.08**
Xanthii Fructus	−94.40	−91.37
Zedoariae Rhizoma	−94.14	−93.79

Supplementary Material Table S2. Changes in the cell density of methicillin-resistant *Staphylococcus aureus* (MRSA) strains MRSA CCARM 3820 and MRSA CCARM 3879 after incubation with oxacillin (1/8 MIC, 4 mg/L) and five selected ethanolic extracts (512 mg/L) for 24 h.

Extract	MRSA CCARM 3820	MRSA CCARM 3879
	Relative cell growth (%)	Relative cell growth (%)
Alpiniae Oxyphyllae Fructus	−34.02	−76.44
**Corydalis Tuber**	**−94.25**	**−96.83**
Lepidii seu Descurainiae Semen	−30.30	−29.78
**Linderae Radix**	**−96.25**	**−95.03**
Thujae Semen	−57.06	−96.53

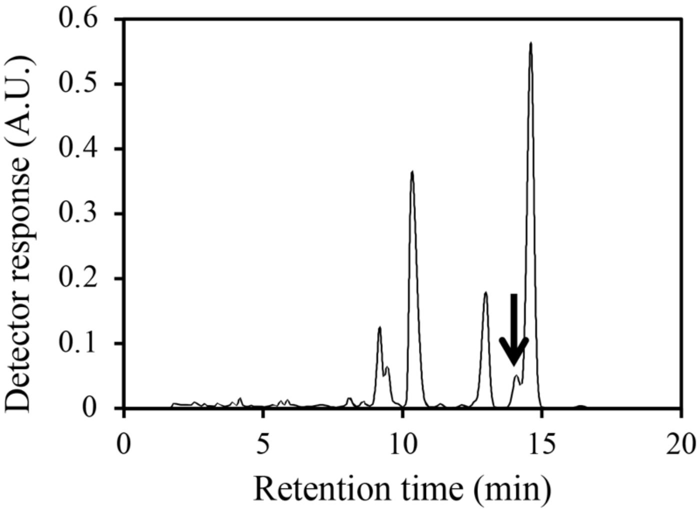

Supplementary Material Figure S2. High-performance liquid chromatography chromatogram of the Corydalis Tuber ethanol extract. The arrow indicates the retention time of berberine.

Supplementary Material Table S3. Simulated molecular docking binding affinities of eight alkaloid compounds known to be in Corydalis Tuber extract to the four efflux pump proteins of *Staphylococcus aureus*.

Name	MepA (kcal/mol)	NorA (kcal/mol)	NorB (kcal/mol)	SdrM (kcal/mol)
Allocrypropin	−6.4	−6.7	−6.4	−6.4
Berberine	−7.9	−7.6	−7.8	−9.4
Coptisine	−7.1	−7.8	−7.1	−7.0
Corydaline	−5.7	−6.1	−5.8	−5.9
Dehyrocorydaline	−6.1	−6.4	−6.0	−6.4
Palmatine	−6.2	−7.0	−5.4	−5.7
Protopine	−6.9	−7.1	−6.8	−7.4
Tetrahydropalmatine	−6.0	−6.1	−6.2	−5.9

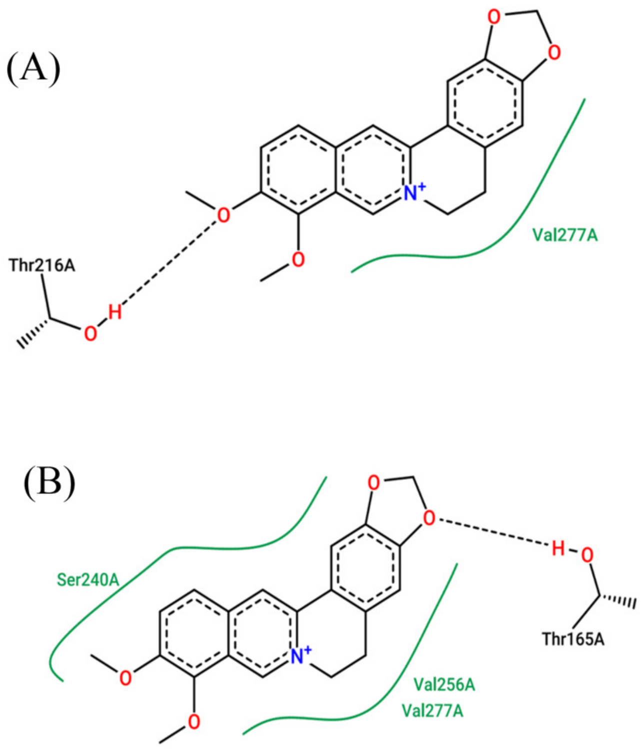

Supplementary Material Figure S3. Molecular docking analysis with berberine and the PBP2A protein of *Staphylococcus aureus* ATCC 33593 (**A**) and *S. aureus* Mu50 (**B**; PDB code: 4CJN). The curved green lines indicate hydrophobic interactions and the black dotted line shows hydrogen bonds.

```
33593   1 MKKIKIVPLI LIVVVVGFGI YFYASKDKEI NNTIDAIEDK NFKQVYKDSS YISKSDNGEV
Mu50    1 MKKIKIVPLI LIVVVVGFGI YFYASKDKEI NNTIDAIEDK NFKQVYKDSS YISKSDNGEV

33593  61 EMTERPIKIY NSLGVKDINI QDRKIKKVSK NKKRVDAQYK IKTNYGNIDR NVQFNFVKED
Mu50   61 EMTERPIKIY NSLGVKDINI QDRKIKKVSK NKKRVDAQYK IKTNYGNIDR NVQFNFVKED

33593 121 GMWKLDWDHS VIIPGMQKDQ SIHIENLKSE RGKILDRNNV ELANTGTAYE IGIVPKNVSK
Mu50  121 GMWKLDWDHS VIIPGMQKDQ SIHIENLKSE RGKILDRNNV ELANTGTAYE IGIVPKNVSK

                                   *
33593 181 KDYKAIAKEL SISEDYIKQQ MDQKWVQDDT FVPLKTVKKM DEYLSDFAKK FHLTTNETES
Mu50  181 KDYKAIAKEL SISEDYIKQQ MDQNWVQDDT FVPLKTVKKM DEYLSDFAKK FHLTTNETES

               *
33593 241 RNYPLEKATS HLLGYVGPIN SEELKQKEYK GYKDDAVIGK KGLEKLYDKK LQHEDGYRVT
Mu50  241 RNYPLGKATS HLLGYVGPIN SEELKQKEYK GYKDDAVIGK KGLEKLYDKK LQHEDGYRVT

33593 301 IVDDNSNTIA HTLIEKKKKD GKDIQLTIDA KVQKSIYNNM KNDYGSGTAI HPQTGELLAL
Mu50  301 IVDDNSNTIA HTLIEKKKKD GKDIQLTIDA KVQKSIYNNM KNDYGSGTAI HPQTGELLAL

33593 361 VSTPSYDVYP FMYGMSNEEY NKLTEDKKEP LLNKFQITTS PGSTQKILTA MIGLNNKTLD
Mu50  361 VSTPSYDVYP FMYGMSNEEY NKLTEDKKEP LLNKFQITTS PGSTQKILTA MIGLNNKTLD

33593 421 DKTSYKIDGK GWQKDKSWGG YNVTRYEVVN GNIDLKQAIE SSDNIFFARV ALELGSKKFE
Mu50  421 DKTSYKIDGK GWQKDKSWGG YNVTRYEVVN GNIDLKQAIE SSDNIFFARV ALELGSKKFE

33593 481 KGMKKLGVGE DIPSDYPFYN AQISNKNLDN EILLADSGYG QGEILINPVQ ILSIYSALEN
Mu50  481 KGMKKLGVGE DIPSDYPFYN AQISNKNLDN EILLADSGYG QGEILINPVQ ILSIYSALEN

33593 541 NGNINAPHLL KDTKNKVWKK NIISKENINL LTDGMQQVVN KTHKEDIYRS YANLIGKSGT
Mu50  541 NGNINAPHLL KDTKNKVWKK NIISKENINL LTDGMQQVVN KTHKEDIYRS YANLIGKSGT

33593 601 AELKMKQGET GRQIGWFISY DKDNPNMMMA INVKDVQDKG MASYNAKISG KVYDELYENG
Mu50  601 AELKMKQGET GRQIGWFISY DKDNPNMMMA INVKDVQDKG MASYNAKISG KVYDELYENG

33593 661 NKKYDIDE
Mu50  661 NKKYDIDE
```

Supplementary Material Figure S4. The PBP2A protein amino acid sequences of *Staphylococcus aureus* ATCC 33593 and *S. aureus* Mu50. An asterisk indicates the presence of different amino acids at that position.
